# The relationship between splenic dose and radiation-induced lymphopenia

**DOI:** 10.1093/jrr/rrae023

**Published:** 2024-05-07

**Authors:** Yifu Ma, Yuehong Kong, Shuying Zhang, Yong Peng, Meiling Xu, Junjun Zhang, Hong Xu, Zhihui Hong, Pengfei Xing, Jianjun Qian, Liyuan Zhang

**Affiliations:** PRaG Therapy Center, Center for Cancer Diagnosis and Treatment, The Second Affiliated Hospital of Soochow University, San Xiang Road No. 1055, Suzhou 215004, China; Department of Radiotherapy and Oncology, The Second Affiliated Hospital of Soochow University, San Xiang Road No. 1055, Suzhou 215004, China; Institute of Radiotherapy and Oncology, Soochow University, San Xiang Road No. 1055, Suzhou 215004, China; PRaG Therapy Center, Center for Cancer Diagnosis and Treatment, The Second Affiliated Hospital of Soochow University, San Xiang Road No. 1055, Suzhou 215004, China; Department of Radiotherapy and Oncology, The Second Affiliated Hospital of Soochow University, San Xiang Road No. 1055, Suzhou 215004, China; Institute of Radiotherapy and Oncology, Soochow University, San Xiang Road No. 1055, Suzhou 215004, China; Department of Ultrasound, The Second Affiliated Hospital of Soochow University, San Xiang Road No. 1055, Suzhou 215004, China; PRaG Therapy Center, Center for Cancer Diagnosis and Treatment, The Second Affiliated Hospital of Soochow University, San Xiang Road No. 1055, Suzhou 215004, China; Department of Radiotherapy and Oncology, The Second Affiliated Hospital of Soochow University, San Xiang Road No. 1055, Suzhou 215004, China; Institute of Radiotherapy and Oncology, Soochow University, San Xiang Road No. 1055, Suzhou 215004, China; PRaG Therapy Center, Center for Cancer Diagnosis and Treatment, The Second Affiliated Hospital of Soochow University, San Xiang Road No. 1055, Suzhou 215004, China; Department of Radiotherapy and Oncology, The Second Affiliated Hospital of Soochow University, San Xiang Road No. 1055, Suzhou 215004, China; Institute of Radiotherapy and Oncology, Soochow University, San Xiang Road No. 1055, Suzhou 215004, China; PRaG Therapy Center, Center for Cancer Diagnosis and Treatment, The Second Affiliated Hospital of Soochow University, San Xiang Road No. 1055, Suzhou 215004, China; Department of Radiotherapy and Oncology, The Second Affiliated Hospital of Soochow University, San Xiang Road No. 1055, Suzhou 215004, China; Institute of Radiotherapy and Oncology, Soochow University, San Xiang Road No. 1055, Suzhou 215004, China; PRaG Therapy Center, Center for Cancer Diagnosis and Treatment, The Second Affiliated Hospital of Soochow University, San Xiang Road No. 1055, Suzhou 215004, China; Department of Oncology, Changshu Hospital Affiliated to Soochow University, Shu Yuan Road No. 1, Suzhou 215500, China; Department of Nuclear medicine, The Second Affiliated Hospital of Soochow University, San Xiang Road No. 1055, Suzhou 215004, China; PRaG Therapy Center, Center for Cancer Diagnosis and Treatment, The Second Affiliated Hospital of Soochow University, San Xiang Road No. 1055, Suzhou 215004, China; Department of Radiotherapy and Oncology, The Second Affiliated Hospital of Soochow University, San Xiang Road No. 1055, Suzhou 215004, China; Institute of Radiotherapy and Oncology, Soochow University, San Xiang Road No. 1055, Suzhou 215004, China; PRaG Therapy Center, Center for Cancer Diagnosis and Treatment, The Second Affiliated Hospital of Soochow University, San Xiang Road No. 1055, Suzhou 215004, China; Department of Radiotherapy and Oncology, The Second Affiliated Hospital of Soochow University, San Xiang Road No. 1055, Suzhou 215004, China; Institute of Radiotherapy and Oncology, Soochow University, San Xiang Road No. 1055, Suzhou 215004, China; PRaG Therapy Center, Center for Cancer Diagnosis and Treatment, The Second Affiliated Hospital of Soochow University, San Xiang Road No. 1055, Suzhou 215004, China; Department of Radiotherapy and Oncology, The Second Affiliated Hospital of Soochow University, San Xiang Road No. 1055, Suzhou 215004, China; Institute of Radiotherapy and Oncology, Soochow University, San Xiang Road No. 1055, Suzhou 215004, China; State Key Laboratory of Radiation Medicine and Protection, Soochow University, Ren Ai Road No. 199, Suzhou 215004, China

**Keywords:** radiotherapy, dose constraints for the spleen, lymphopenia

## Abstract

Lymphocytes, which are highly sensitive to radiation, play a crucial role in the body’s defense against tumors. Radiation-induced lymphopenia has been associated with poorer outcomes in different cancer types. Despite being the largest secondary lymphoid organ, the spleen has not been officially designated as an organ at risk. This study hypothesizes a connection between spleen irradiation and lymphopenia and seeks to establish evidence-based dosage limits for the spleen. We retrospectively analyzed data from 96 patients with locally advanced gastric cancer who received postoperative chemoradiotherapy (CRT) between May 2010 and May 2017. Complete blood counts were collected before, during and after CRT. We established a model for predicting the minimum absolute lymphocyte count (Min ALC) and to investigate potential associations between spleen dosimetric variables and Min ALC. The median follow-up was 60 months. The 5-year overall survival (OS) and disease-free survival (DFS) were 65.2% and 56.8%, respectively. The median values of pre-treatment ALC, Min ALC and post-treatment ALC were 1.40 × 10^9^, 0.23 × 10^9^ and 0.28 × 10^9^/L, respectively. Regression analysis confirmed that the primary tumor location, number of fractions and spleen V5 were significant predictors of Min ALC during radiation therapy. Changes in ALC (ΔALC) were identified as an independent predictor of both OS and DFS. Spleen V5 is an independent predictor for Min ALC, and the maximum dose of the spleen is associated with an increased risk of severe lymphopenia. Therefore, these doses should be restricted in clinical practice. Additionally, ΔALC can serve as a prognostic indicator for adjuvant radiotherapy in gastric cancer.

## INTRODUCTION

Lymphocytes play a crucial role in fighting against tumors, especially in the age of immune checkpoint inhibitors. They are highly sensitive to radiation, with an LD90 (a lethal dose reducing survival by 90%) of 3 Gy, making them vulnerable to depletion even at low radiation doses (<1 Gy) [1]. Accumulating studies have demonstrated that a decrease in absolute lymphocyte count (ALC) might be correlated with the total dose delivered and the total number of circulating lymphocytes exposed to radiotherapy [2, 3]. Lymphopenia is a well-established complication of radiotherapy that is associated with poor prognosis. Past studies indicated that radiation-induced lymphopenia (RIL) was associated with unfavorable patient prognosis in different cancers [4–6]. However, the spleen, a significant secondary lymphoid organ with extensive blood supply, has not been identified as an organ at risk (OAR), and there are no recommended radiation dose limits for it in abdominal treatments, despite its frequent exposure [7–9]. Consequently, optimizing the delineation of the radiation target and limiting the radiation dose to the spleen in abdominal radiotherapy may be crucial for preserving circulating lymphocytes.

Leukopenia, which includes neutropenia and lymphopenia, is a common side effect during radiation therapy, and poses potential life-threatening risks if severe. Therefore, close monitoring of leukopenia during radiotherapy is crucial. There is a strong association between spleen dose and leukopenia [6]. However, with the development of immunotherapy, several studies have linked RIL to unfavorable outcomes [10, 11]. Our study focuses on the relationship between spleen dose and RIL. In contrast to previous studies, this retrospective study is one of the few to focus on patients with locally advanced gastric cancer who received adjuvant radiotherapy. In postoperative chemoradiotherapy (CRT) for gastric cancer, the clinical target volume (CTV) typically includes the tumor bed, anastomosis site and selected regional lymph nodes (LNs) [12]. This broad treatment area in the upper abdomen may expose neighboring organs, such as the spleen, to potential harm.

In this context, our study seeks to explore the connection between spleen radiation parameters and the risk of lymphopenia in patients undergoing postoperative CRT for gastric cancer. We also assess the relationship between RIL and patient prognosis, with the goal of developing a predictive model for RIL.

## MATERIALS AND METHODS

### Patients

This retrospective study included patients who had undergone radical resections for gastric cancer at our hospital between May 2010 and May 2017. Inclusion criteria were (i) R0 gastrectomy and D1+ lymphadenectomy or higher; (ii) no clinical signs of distant or peritoneal metastasis; (iii) receipt of postoperative CRT; (iv) regular post-treatment follow-ups; (v) comprehensive medical records. Exclusion criteria were (i) non-radical surgery; (ii) distant metastasis before surgery; (iii) palliative or preoperative therapy; (iv) incomplete medical records; (v) history of splenectomy, splenomegaly or inadequate function of vital organs like the liver or kidneys.

### Treatment

Intensity-modulated radiation therapy (IMRT) was used for radiotherapy. Patients were treated with a median dose of 45 Gy (range, 41.4–50.4 Gy) delivered at 1.8 Gy/fraction. Radiation target volumes included the tumor bed, anastomosis site and selected regional LNs (Supplementary Fig. 1). Patients with pT1–3 gastric cancer did not receive radiation to the tumor bed. The choice of regional LNs, such as perigastric, celiac, splenic, hepatoduodenal, hepatic portal, pancreaticoduodenal and paraaortic LNs, depended on the tumor’s location (see Supplementary Table S1). The radiation dose constraints were spinal cord Dmax ≤45 Gy, kidney V20 < 25%, liver V30 < 30% and heart V30 < 30%. No dose constraints were applied to the spleen.

A radiation oncologist outlined the spleen for each patient, and its dosimetry was calculated and approved using the Pinnacle treatment plan by a medical physicist. Spleen dosimetric parameters, comprising the maximum dose (Dmax), the mean dose (Dmean) and various volumetric proportions of the spleen receiving ≥ *x* Gy (V*x*), were extracted from the treatment plan.

### Clinical data and assessment of absolute peripheral lymphocytes

Comprehensive clinical data were collected from enrolled patients, including age, sex, pathologic types, Lauren’s classification, operative approach, primary tumor location, lymphovascular invasion (LVI), perineural invasion (PNI), pathologic tumor-node-metastasis (pTNM) stage, concurrent and adjuvant chemotherapy regimens and blood test results.

The pre-treatment count was defined as the most recent blood count before the start of radiotherapy, while the post-treatment count was defined as the most recent blood count after completing radiotherapy. Minimum absolute lymphocyte count (Min ALC) was determined as the minimum value of ALCs in peripheral blood during radiotherapy. Pre-treatment ALC, pre-treatment absolute neutrophil count (ANC), Min ALC, post-treatment ALC and post-treatment ANC were recorded. ΔALC was calculated by subtracting pre-treatment ALC from Min ALC. The neutrophil-to-lymphocyte ratio (NLR) was represented by the ratio between ANC and ALC. The lymph node ratio (LNR) was determined by dividing the total number of metastatic LNs by the total number of examined LNs. Adverse events were assessed according to Common Terminology Criteria for Adverse Events version 5.0.

### Follow-up

Following adjuvant CRT completion, regular follow-up assessments were conducted in accordance with the institutional surveillance protocol. These assessments included medical history reviews, physical examinations, serum biochemical tests, tumor biomarker evaluations, chest, abdomen and pelvis CT scans (or positron-emission tomographic scans if necessary) and endoscopy at each visit. Patients were followed up every 3 months for the initial 2 years, every 6 months up to 5 years and annually thereafter.

### Statistical analysis

Patient characteristics were assessed using descriptive statistics. The association with blood counts (Min ALC, post-treatment ALC and post-treatment ANC) and the following dependent variables were individually tested with univariate simple linear regression analysis. These variables included age, primary tumor location, pathologic types, Lauren’s classification, LNR, pTNM stage, LVI, PNI, concurrent chemotherapy regimen, adjuvant chemotherapy regimen, pre-treatment count, integral planning target volume dose, number of fractions, Dmean of the spleen, Dmax of the spleen and volumetric proportions of the spleen (V5, V10, V15, V20, V25, V30, V35, V40, V45). Multiple stepwise linear regression analysis was conducted for significant variables identified in the univariate analysis to establish a predictive model for blood counts during and after treatment. To assess prognostic impact, time-to-event analysis was performed for overall survival (OS) and disease-free survival (DFS) using the Kaplan–Meier method. Univariate and multivariate analyses were executed using Cox regression analysis for the following variables: sex, age, primary tumor location, pathologic types, Lauren’s classification, LNR, pTNM stage, LVI, PNI, concurrent chemotherapy regimen, adjuvant chemotherapy regimen, blood count, ΔALC and NLR. Survival plots for ΔALC and NLR, dichotomized by median, were created using the Kaplan–Meier method. Nonparametric tests were utilized to compare the following parameters between individuals who developed grade 3–4 lymphopenia and those who did not: age, dose per fraction, number of fractions, pre-treatment ALC, Dmean of the spleen, Dmax of the spleen and volumetric proportions of the spleen (V5, V10, V15, V20, V25, V30, V35, V40, V45). Receiver operating characteristic curve analysis was carried out to determine the cutoff value of Dmax of the spleen in predicting the development of grade 3–4 lymphopenia. All statistical analyses were performed using IBM SPSS v. 25.0 (SPSS Inc., Chicago, IL), and statistical significance was considered at a *P* value <0.05 from two-sided tests.

## RESULTS

**Figure 1:**
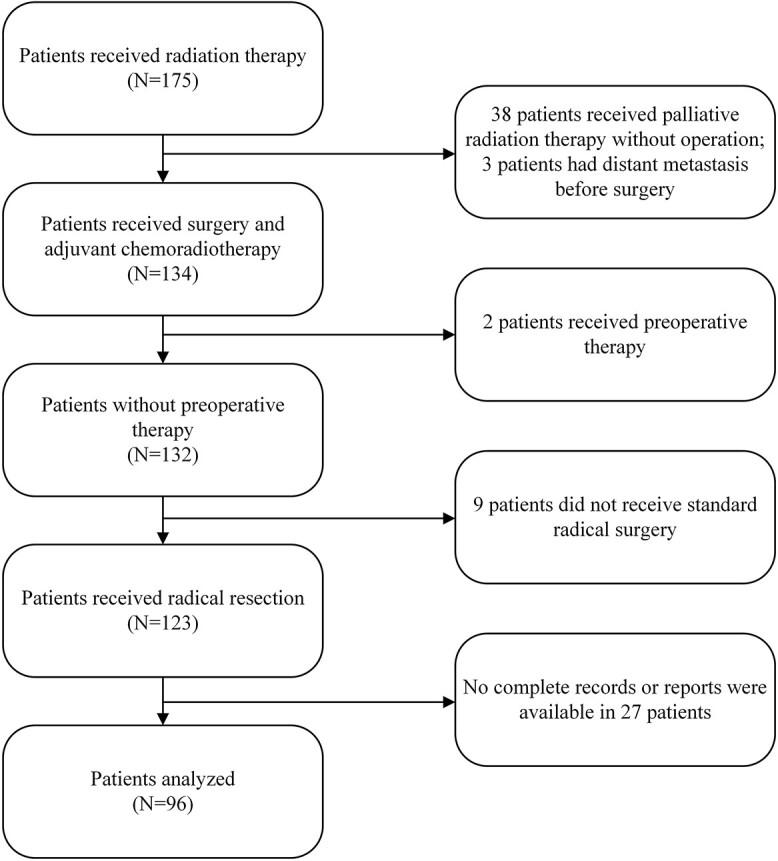
Flow diagram of patient selection according to the eligibility criteria and exclusion criteria.

Out of the total of 175 gastric cancer patients who received radiotherapy for abdominal tumors, 79 were excluded for the following reasons: 38 received palliative radiation therapy without surgery, three had distant metastasis before gastrectomy, two received preoperative therapy, nine did not undergo standard radical surgery and 27 had incomplete medical records. In the end, 96 patients met the criteria and were included in the analysis (see Fig. 1). The first patient underwent radical surgery in July 2010, and the last patient was treated in April 2017. The median duration between the surgery and the nearest blood draw before radiotherapy was 73.5 days (range, 50–126 days). The loss to follow-up rate was 7.3% (7 out of 96 patients).

**Figure 2:**
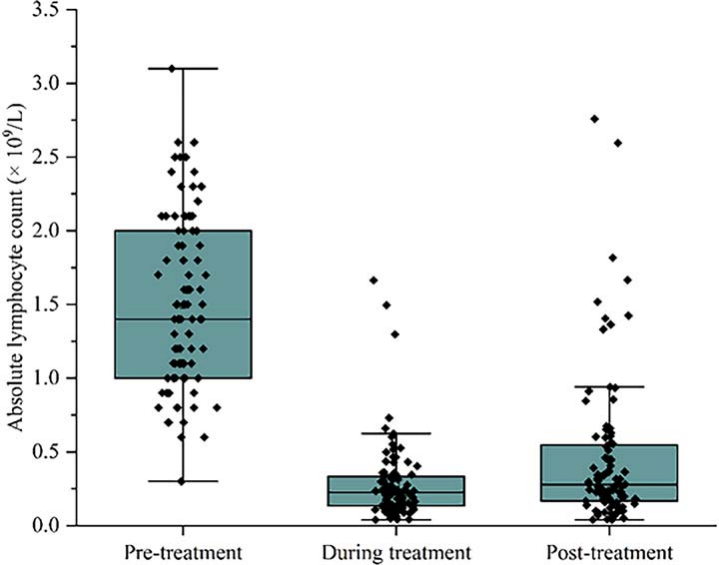
The kinetics of lymphocyte counts during radiotherapy. The median values (×10^9^ cells/L) of pre-treatment ALC, min ALC and post-treatment ALC are 1.40 (range, 0.30–3.10), 0.23 (range, 0.04–1.66) and 0.28 (range, 0.04–2.76), respectively.

**Figure 3:**
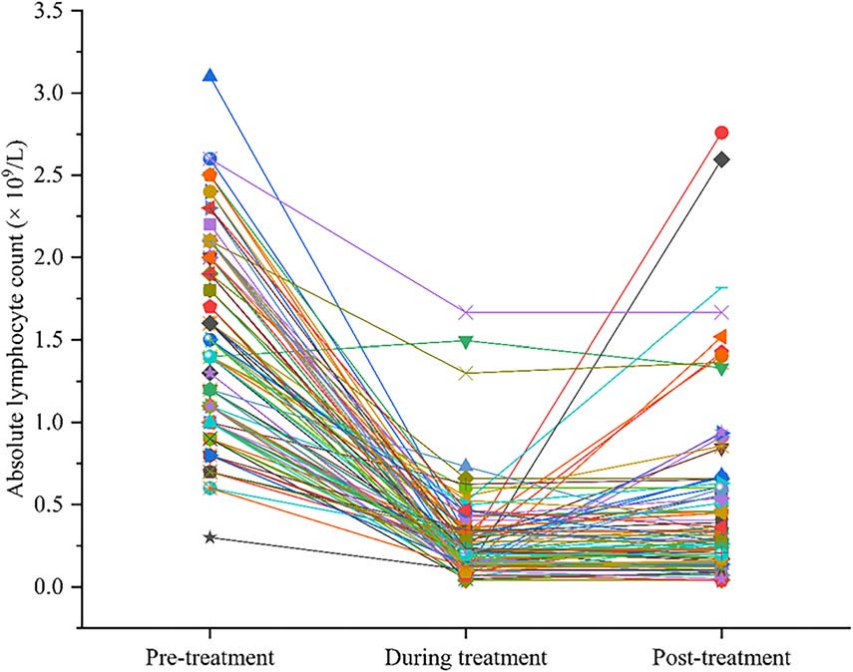
ALC trend of 96 gastric cancer patients who received postoperative CRT.

The study cohort consisted of 30 patients with pathological stage II and 66 patients with pathological stage III (American Joint Committee on Cancer, eighth edition) who received IMRT and concurrent chemotherapy after radical gastrectomy. The clinicopathological data, dosimetric data and blood count data for the entire cohort are presented in Table 1. Among the 96 patients, 75.0% were male, and the median age was 60 years (range, 27–72 years). Of the enrolled patients, 68.8% were in pathological stage III, and 38.5% had N3 disease. The median values (×10^9^) for pre-treatment ALC, Min ALC and post-treatment ALC were 1.40 (range, 0.30–3.10), 0.23 (range, 0.04–1.66) and 0.28 (range, 0.04–2.76), respectively. The average duration from the start of CRT to the observed minimum ALC was 27.9 days. The median values of ALC decreased by 80% after postoperative CRT. The median duration from the last day of CRT to the nearest subsequent blood draw was 7 days (range, 0–43 days). To visualize the trends of peripheral blood lymphocytes during radiotherapy, ALC values were plotted against time during radiotherapy (see Figs 2 and 3).

**Table 1:** Baseline demographic, tumor and treatment characteristics of 96 gastric cancer patients

Characteristic	n (%) or median (range)
Sex	
Male	72 (75.0%)
Female	24 (25.0%)
Age (years)	60 (27–72)
Operative hospital	
Our hospital	59 (61.5%)
Another hospital	37 (38.5%)
Operative approach	
Total gastrectomy	34 (35.4%)
Subtotal gastrectomy	57 (59.4%)
Gastrectomy combined with resection of other organs	5 (5.2%)
Primary tumor location	
Upper 1/3	23 (24.0%)
Middle 1/3	19 (19.8%)
Lower 1/3	47 (49.0%)
Total stomach	7 (7.2%)
Pathologic types	
Well to moderately differentiated adenocarcinoma	34 (35.4%)
Poorly differentiated adenocarcinoma	55 (57.3%)
Others	7 (7.3%)
Lauren’s classification	
Intestinal type	39 (40.6%)
Diffuse type	52 (54.2%)
Others	5 (5.2%)
LN status	
No. of dissected LNs, median (range)	18 (6–56)
No. of positive LNs, median (range)	5 (0–30)
LNR = 0	15 (15.6%)
0 < LNR < 0.3	38 (39.6%)
0.3 ≤ LNR < 0.7	37 (38.5%)
0.7 ≤ LNR	6 (6.3%)
Pathologic T stage	
T1	6 (6.3%)
T2	11 (11.5%)
T3	43 (44.8%)
T4	36 (37.5%)
Pathologic N stage	
N0	14 (14.6%)
N1	17 (17.7%)
N2	28 (29.2%)
N3	37 (38.5%)
Stage^a^	
IIA	18 (18.8%)
IIB	12 (12.5%)
IIIA	28 (29.2%)
IIIB	24 (25.0%)
IIIC	14 (14.6%)
Lymphovascular invasion	
Negative	49 (51%)
Positive	47 (49%)
Perineural invasion	
Negative	52 (54.2%)
Positive	44 (45.8%)
Concurrent chemotherapy regimen	
Capecitabine	61 (63.5%)
Tegafur/Gimeracil/Oteracil	15 (15.6%)
Others	20 (20.9%)
Adjuvant chemotherapy regimen	
CAPOX	50 (52.1%)
SOX	13 (13.5%)
FLOFOX	9 (9.4%)
EOF	6 (6.3%)
Others	18 (18.7%)
Hematologic toxicity^b^	
Grade 3 lymphopenia	44 (45.8%)
Grade 4 lymphopenia	42 (43.8%)
Grade 3 leukopenia	15(15.6%)
Grade 4 leukopenia	2 (2.1%)
Spleen dosimetric data	
Dmean of Spleen	2774 (398–3962)
Dmax of Spleen	4878 (2010–5483)
Proportion of spleen volume receiving at least:	
5Gy (V5), median (range)	99.91 (23.41–100)
10Gy (V10), median (range)	91.29 (5.25–100)
15Gy (V15), median (range)	80.37 (2.04–100)
20Gy (V20), median (range)	69.53 (0–100)
25Gy (V25), median (range)	56.73 (0–100)
30Gy (V30), median (range)	43.30 (0–97.28)
35Gy (V35), median (range)	29.75 (0–81.11)
40Gy (V40), median (range)	19.61 (0–59.65)
45Gy (V45), median (range)	8.29 (0–45.39)
Blood count data^c^	
ALC before radiotherapy	1.40 (0.30–3.10)
ALC during radiotherapy	0.23 (0.04–1.66)
ALC after radiotherapy	0.28 (0.04–2.76)
ANC before radiotherapy	2.70 (0.40–15.20)
ANC during radiotherapy	2.34 (0.12–15.84)
ANC after radiotherapy	2.07 (0.40–6.22)

Abbreviations: LNs = lymph nodes, LNR = lymph node ratio, CAPOX = capecitabine and oxaliplatin, SOX = Tegafur/Gimeracil/Oteracil and oxaliplatin, FOLFOX = folinic acid, fluorouracil, and oxaliplatin, EOF = epirubicin, oxaliplatin, and fluorouracil, ALC = absolute lymphocyte count, ANC = absolute neutrophil count.

^a^American Joint Committee on Cancer, eighth edition.

^b^Common Terminology Criteria for Adverse Events 5.0.

^c^= 10^9^ cells/L

Patients received a median radiation dose of 45 Gy (range, 41.4–50.4 Gy), delivered in daily fractions of 1.8 Gy. The median duration of radiation treatment was 35 days (range, 30–45 days). Chemotherapy was administered 3–8 weeks after surgery, followed by chemoradiation beginning 8–18 weeks after surgery. The concurrent chemotherapy regimens included capecitabine (61, 63.5%), Tegafur/Gimeracil/Oteracil (15, 15.6%) and other agents (20, 20.9%). All enrolled patients received adjuvant chemotherapy before or after radiotherapy, with chemotherapy regimens comprising CAPOX (capecitabine and oxaliplatin) (50, 52.1%); SOX (Tegafur/Gimeracil/Oteracil and oxaliplatin) (13, 13.5%); FOLFOX (folinic acid, fluorouracil and oxaliplatin) (9, 9.4%); EOF (epirubicin, oxaliplatin and fluorouracil) (6, 6.3%); and other agents (18, 18.7%).

**Figure 4:**
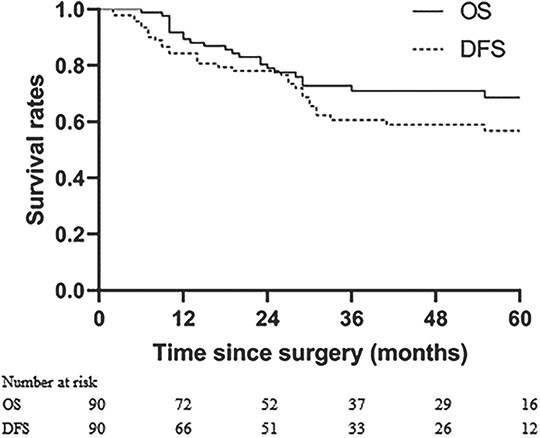
Kaplan–Meier estimate of OS and DFS.

**Figure 5:**
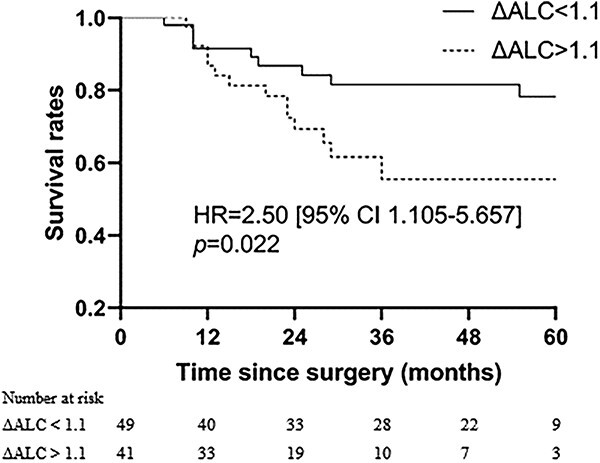
Overall survival with ΔALC dichotomized by median. ΔALC = the difference between pre-treatment ALC and minimum ALC during radiotherapy.

**Figure 6:**
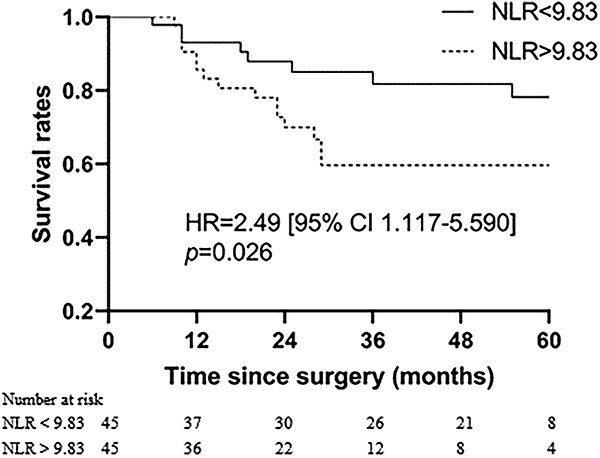
Overall survival with NLR dichotomized by median.

**Figure 7:**
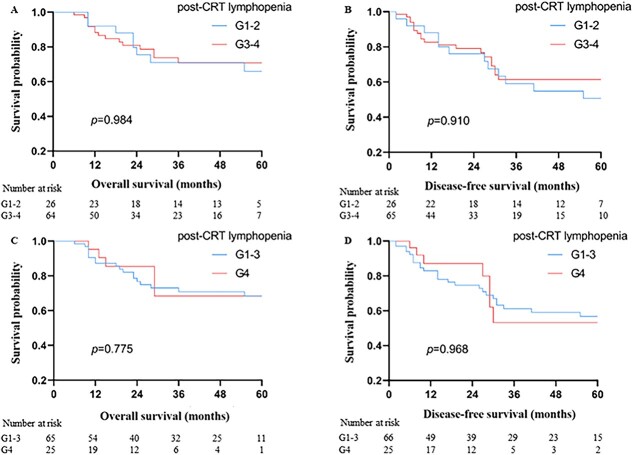
Kaplan–Meier curves show OS (A and C), DFS (B and D) of gastric cancer patients, grouped by lymphopenia grade (Common Terminology Criteria for Adverse Events 5.0) based on ALC post CRT.

The patients were observed until May 2020, and the median follow-up period spanned 60.0 months (range, 36–110 months). The 1-, 3- and 5-year OS were 89.4%, 70.9% and 65.2%, respectively, while the 1-, 3- and 5-year DFS were 84.3%, 60.6% and 56.8% (refer to Fig. 4). In univariate analysis, factors related to OS included pathologic types, Lauren’s classification, LNR, pTNM stage, concurrent chemotherapy regimen, adjuvant chemotherapy regimen, ΔALC and NLR. Further multivariate analysis revealed that LNR, adjuvant chemotherapy regimen and ΔALC were independent prognostic factors (consult Table 2). Regarding DFS, pathologic types, Lauren’s classification, concurrent chemotherapy regimen, ΔALC and NLR were associated with the outcome. Notably, multivariate analysis showed that ΔALC was the sole independent prognostic factor linked to DFS (see Table 3). Survival plots for ΔALC and NLR, dichotomized by the median, are displayed in Figs 5 and 6, respectively. Patients with ΔALC <1.1 × 10^9^ cell/L exhibited longer OS compared with those with ΔALC ≥1.1 × 10^9^ cell/L (HR = 2.50, *P* = 0.022). Additionally, patients with NLR ≥ 9.83 had a shorter OS duration compared with those with NLR < 9.83 (HR = 2.49, *P* = 0.026). No significant differences were observed in OS and DFS between patients with grade 3–4 RIL and grade 1–2 RIL. Similarly, there were no significant differences in OS and DFS between patients with grade 4 RIL and grade 1–3 RIL (see Fig. 7).

**Table 2:** Univariate and multivariate analyses of overall survival

Variable	Univariate		Multivariate	
	HR [95%CI]	*P*	HR [95%CI]	*P*
Sex		0.130		
Age		0.356		
Primary tumor location		0.345		
Pathologic types	4.966 [1.240–19.892]	**0.024**		
Lauren’s classification	3.793 [1.295–11.113]	**0.015**		
Lymph node ratio	1.763 [1.092–2.845]	**0.020**	1.991 [1.243–3.190]	**0.004**
Stage^a^	1.413 [1.023–1.951]	**0.036**		
Lymphovascular invasion		0.558		
Perineural invasion		0.346		
Concurrent chemotherapy regimen	3.492 [1.315–9.271]	**0.012**		
Adjuvant chemotherapy regimen	4.641 [1.651–13.049]	**0.004**	4.620 [1.439–14.836]	**0.010**
ALC before RT		0.085		
ALC during RT		0.872		
ALC after RT		0.785		
ANC before RT		0.998		
ANC after RT		0.723		
ΔALC^b^	2.557 [1.107–5.908]	**0.028**	2.582 [1.037–6.431]	**0.042**
NLR	2.565 [1.084–6.071]	**0.032**		

Abbreviations: CI = confidence interval, HR = hazard ratio, ALC = absolute lymphocyte count, RT = radiotherapy, ANC = absolute neutrophil count, NLR = neutrophil-to-lymphocyte ratio.

^a^American Joint Committee on Cancer, eighth edition.

^b^the difference between pre-treatment ALC and minimum ALC during radiotherapy.

Statistically significant values are highlighted in bold.

**Table 3:** Univariate and multivariate analyses of disease-free survival

Variable	Univariate		Multivariate	
	HR [95%CI]	*P*	HR [95%CI]	*P*
Sex		0.558		
Age		0.671		
Primary tumor location		0.416		
Pathologic types	2.434 [1.138–5.207]	**0.022**		
Lauren’s classification	3.069 [1.471–6.402]	**0.003**		
Lymph node ratio		0.069		
Stage^a^		0.111		
Lymphovascular invasion		0.361		
Perineural invasion		0.850		
Concurrent chemotherapy regimen	3.895 [1.637–9.268]	**0.002**		
Adjuvant chemotherapy regimen		0.071		
ALC before RT		0.079		
ALC during RT		0.277		
ALC after RT		0.579		
ANC before RT		0.511		
ANC after RT		0.733		
ΔALC^b^	2.720 [1.416–5.223]	**0.003**	2.800 [1.446–5.424]	**0.002**
NLR	2.055 [1.089–3.877]	**0.026**		

Abbreviations: CI = confidence interval, HR = hazard ratio, ALC = absolute lymphocyte count, RT = radiotherapy, ANC = absolute neutrophil count, NLR = neutrophil-to-lymphocyte ratio.

^a^American Joint Committee on Cancer, eighth edition.

^b^the difference between pre-treatment ALC and minimum ALC during radiotherapy.

Statistically significant values are highlighted in bold.

The results of the simple linear and multiple stepwise linear regression analyses investigating the correlation with Min ALC are detailed in Table 4. On univariate simple linear regression, primary tumor location, pTNM stage, LVI, number of fractions, Dmean of the spleen, spleen V5, V10, V15, V20 and V30 were all significantly associated with Min ALC during radiotherapy. However, in multiple stepwise linear regression analysis, primary tumor location (β = 0.254, *P* = 0.005), number of fractions (β = −0.250, *P* = 0.008) and spleen V5 (β = −0.348, *P* < 0.001) were identified as significant factors associated with Min ALC during radiotherapy (R^2^ = 0.298, F = 13.043; *P* < 0.001). Additionally, we found that spleen V5 (β = −0.282, *P* = 0.005) was the only significant factor associated with post-treatment ANC (R^2^ = 0.080, F = 8.142; *P* = 0.005). Our study did not identify any significant factors associated with post-treatment ALC (refer to Table 5).

**Table 4:** Regression analysis of predictor variables of ALC during radiotherapy

Variable	ALC during radiotherapy			
	Univariate		Multivariate	
	β	*P*	β	*P*
Age	-	0.289		
Primary tumor location	**0.238**	**0.019**	**0.254**	**0.005**
Pathologic types	-	0.413		
Lauren’s classification	-	0.388		
Lymph node ratio	-	0.126		
Stage^a^	**−0.202**	**0.049**		
Lymphovascular invasion	**−0.208**	**0.042**		
Perineural invasion	-	0.051		
Concurrent chemotherapy regimen	-	0.077		
Adjuvant chemotherapy regimen	-	0.982		
Pre-treatment count	-	0.072		
Integral PTV dose	-	0.173		
Number of fractions	**−0.368**	**0.000**	**−0.250**	**0.008**
Dmean of spleen	**−0.272**	**0.007**		
Dmax of spleen	-	0.103		
Spleen V5^b^	**−0.418**	**0.000**	**−0.348**	**0.000**
Spleen V10	**−0.337**	**0.001**		
Spleen V15	**−0.304**	**0.003**		
Spleen V20	**−0.249**	**0.014**		
Spleen V25	-	0.051		
Spleen V30	**−0.224**	**0.028**		
Spleen V35	-	0.071		
Spleen V40	-	0.104		
Spleen V45	-	0.186		

Abbreviations: ALC = absolute lymphocyte count, PTV = planning target volume, β = standardized coefficient.

^a^American Joint Committee on Cancer, eighth edition.

^b^Spleen V*x*: proportion of spleen volume receiving at least *x* Gy.

Statistically significant values are highlighted in bold.

**Table 5:** Regression analysis of predictor variables of ALC post radiotherapy and ANC post radiotherapy

Variable	Post-treatment ALC				Post-treatment ANC			
	Univariate		Multivariate		Univariate		Multivariate	
	β	*P*	β	*P*	β	*P*	β	*P*
Age	-	0.611			-	0.751		
Primary tumor location	-	0.468			-	0.162		
Pathologic types	-	0.780			-	0.411		
Lauren’s classification	-	0.795			-	0.361		
Lymph node ratio	-	0.442			-	0.204		
Stage^a^	-	0.521			-	0.054		
Lymphovascular invasion	-	0.755			-	0.513		
Perineural invasion	-	0.139			-	0.461		
Concurrent chemotherapy regimen	-	0.649			-	0.318		
Adjuvant chemotherapy regimen	-	0.419			-	0.097		
Pre-treatment count	-	0.593			-	0.905		
Integral PTV dose	-	0.393			-	0.778		
Number of fractions	-	0.759			**−0.265**	**0.009**		
Dmean of spleen	-	0.235			-	0.151		
Dmax of spleen	-	0.683			-	0.175		
Spleen V5^b^	-	0.067			**−0.282**	**0.005**	**−0.282**	**0.005**
Spleen V10	-	0.157			-	0.110		
Spleen V15	-	0.233			-	0.129		
Spleen V20	-	0.121			-	0.114		
Spleen V25	-	0.271			-	0.160		
Spleen V30	-	0.254			-	0.381		
Spleen V35	-	0.423			-	0.616		
Spleen V40	-	0.586			-	0.736		
Spleen V45	-	0.975			-	0.234		

Abbreviations: ALC = absolute lymphocyte count, ANC = absolute neutrophil count, PTV = planning target volume, β = standardized coefficient.

^a^American Joint Committee on Cancer, eighth edition.

^b^Spleen V*x*: proportion of spleen volume receiving at least *x* Gy.

Statistically significant values are highlighted in bold.

Only one patient experienced radiation interruption or incomplete radiation. Among all patients who received concurrent chemotherapy, 16 (16.7%) individuals encountered dose delay or reduction. The incidence of grade ≥3 leukopenia and lymphopenia were 17.7% and 89.6%, respectively. The incidence of grade 4 leukopenia and lymphopenia were 2.1% and 43.8%, respectively. Dmax of the spleen was the sole significant factor associated with grade ≥3 lymphopenia (see Table 6). And we found no effect of spine dose on lymphopenia in our study (Supplementary Table S2).

**Table 6:** Median values for patient characteristics and dose–volume parameters in those who developed ≥grade 3 lymphopenia^a^

Median	≥Grade 3 (*n* = 86)	<Grade 3 (*n* = 10)	*P*
Age	60 (27–72)	57 (32–66)	0.175
Dose per fraction (Gy)	1.8 (1.6–2.0)	1.8 (1.8–2.0)	0.530
Number of fractions	25 (23–28)	25 (22–25)	0.108
Pre-treatment ALC	1.4 (0.3–3.1)	1.4 (1.0–2.6)	0.556
Dmean of spleen	2786.5 (603.0–3962.0)	2567.0 (398.0–3361.0)	0.381
Dmax of spleen	4887.0 (2010.0–5483.0)	4793.0 (4036.0–4860.0)	**0.002**
Spleen V5^b^	99.91 (65.52–100.00)	99.21 (23.41–100.00)	0.699
Spleen V10	92.07 (5.25–100.00)	84.18 (7.32–100.00)	0.267
Spleen V15	80.69 (2.04–100.00)	76.80 (3.27–99.11)	0.422
Spleen V20	69.53 (0.00–100.00)	70.20 (0.62–95.69)	0.746
Spleen V25	56.73 (0.00–100.00)	55.04 (0.33–77.19)	0.666
Spleen V30	43.52 (0.00–97.28)	33.67 (0.16–66.54)	0.394
Spleen V35	29.84 (0.00–81.11)	23.82 (0.06–57.82)	0.408
Spleen V40	19.97 (0.00–59.65)	16.29 (0.00–36.76)	0.250
Spleen V45	9.01 (0.00–45.39)	5.07 (0.00–18.98)	0.115

Abbreviations: ALC = absolute lymphocyte count.

^a^Common Terminology Criteria for Adverse Events 5.0.

^b^Spleen V*x*: proportion of spleen volume receiving at least *x* Gy.

Statistically significant values are highlighted in bold.

## DISCUSSION

Radiotherapy plays a crucial role in the development of treatment-related lymphopenia. A matched analysis involving 480 patients with esophageal cancer undergoing neoadjuvant CRT, whether through intensity-modulated or proton beam therapy, revealed no significant reduction in ALC during induction chemotherapy. However, a notable decrease was observed during concurrent CRT [13]. In a study involving 47 patients with stage III non-small cell lung cancer, neoadjuvant chemotherapy did not result in the emergence of lymphopenia. Nevertheless, the mean ALC dropped by 67%, and nearly half of the patients exhibited grade 3 or 4 lymphopenia following radiotherapy [14].

The origin of RIL may be attributed to the irradiation of circulating blood. Severe RIL can be induced by irradiating circulating blood with a built-in radiation source in hemodialysis machines [15]. After radiation to certain organs with limited bone marrow and lymphoid tissue but rich blood supply, there is also a decrease in peripheral blood lymphocyte count [16]. Circulating T lymphocytes play a crucial role in suppressing tumor development and progression [17]. An increasing body of evidence suggests a link between RIL and poor prognosis [18], possibly due to the opportunity it provides for residual tumor cell repopulation after radiotherapy. Our analysis revealed that ΔALC was the only independent prognostic factor associated with OS and DFS. A meta-analysis established a statistically significant association between RIL and OS, showing a 65% increased risk of mortality in patients with grade ≥3 RIL compared with those with grade 0–2 RIL and a 50% surge in mortality in patients with grade 4 RIL compared with grade 0–3 RIL [19]. Another retrospective study of esophageal cancer patients undergoing neoadjuvant CRT found that grade 4 lymphopenia was significantly linked to reduced progression-free survival (*P* = 0.037) and distant metastasis-free survival (*P* = 0.026) in comparison with grade 1–3 [13]. Furthermore, several studies have indicated that grade 3–4 lymphopenia may predict worse DFS [20–26]. Therefore, lymphocyte-rich organs like the spleen should be considered as OARs to enhance the prognosis of cancer patients.

As the largest secondary lymphoid organ, the spleen has an abundant blood supply and plays a vital role in immune response regulation and blood filtration [9]. A significant number of lymphocytes pass through the splenic red pulp structure daily [27]. RIL is likely attributed to both circulating lymphocytes and the splenic pool exposed to radiotherapy [3, 23, 28, 29]. Consequently, higher doses delivered to the spleen increase the risk of severe post-CRT lymphopenia. In a study involving 177 pancreatic cancer patients treated with induction chemotherapy followed by concurrent CRT, median Dmean, V10, V15 and V20 of the spleen were significantly higher in patients with severe lymphopenia compared with those without severe lymphopenia [30]. The Dmean threshold for the development of grade 3–4 lymphopenia was 9 Gy. In our study, the threshold was 18.53 Gy, likely due to variations in target volumes between pancreatic and gastric cancers.

A growing body of evidence highlights a significant correlation between higher spleen dose–volume parameters and the development of severe lymphopenia during CRT. For instance, in a study involving 59 hepatocellular carcinoma patients who received conventional RT with curative intent up to 50–60 Gy, V5 of the spleen emerged as the sole independent predictor for reduced lymphocyte counts [4]. Similarly, a retrospective study of 61 esophageal cancer patients treated with definitive CRT revealed that V5, V10, V20, V30 of the spleen, and the mean splenic dose were significant independent factors negatively influencing the nadir of lymphocyte counts [5]. Our investigation established that the mean splenic dose, V5, V10, V15, V20 and V30 were associated with the nadir of lymphocyte counts, with V5 standing out as a significant predictor of this nadir during treatment according to multivariate regression analysis.

Lee *et al*. [31] found that the mean splenic dose was a significant predictor of lymphopenia. This difference may be that in Lee *et al*.’s study, the severe lymphopenia was assessed at the first post-CRT blood test (the median time was 3 weeks after completion of adjuvant CRT, with interquartile range 1–4 weeks), whereas in our study we investigate the Min ALC during radiotherapy. Furthermore, the majority of patients (72.6%) in Lee *et al*.’s study received three-dimensional conformal radiotherapy, while in our study all patients received IMRT. And the mean splenic dose of patients in Lee *et al*.’s study (40.7 Gy) was higher than that in our study (27.74 Gy), and median V5 in Lee *et al*.’s study was 100%, so it was not possible to use V5 to predict lymphopenia.

Moreover, Dmax of the spleen was identified as a predictor of grade ≥3 lymphopenia. Consequently, we recommend adhering to the ‘As Low As Reasonably Achievable’ principle for lymphocyte-rich organs [32], suggesting that spleen-sparing radiotherapy should be considered to reduce the risk of severe lymphopenia and enhance long-term survival [18]. As a result, we propose that clinical practice should limit spleen V5.

In our study, the mean splenic dose was 27.74 Gy, which contrasts with the findings of Chadha *et al*. [30] in their study of 177 pancreatic cancer patients (6.8 Gy) and Chin *et al*. [6] in their study of 60 patients with distal esophageal and gastroesophageal junction cancer (23.1 Gy). This discrepancy may be attributed to two reasons. Firstly, differences in the CTV resulted from different tumor primary sites. The distance of the CTV from the spleen was a direct factor affecting the splenic dose. In the study by Chadha *et al*., 60% of patients had tumors located in the head of the pancreas, and 7% had tumors in the tail of the pancreas. All patients in our study were locally advanced gastric cancer who underwent adjuvant radiotherapy. Secondly, there are differences in radiotherapy techniques, as some patients in Chadha *et al*.’s study received three-dimensional conformal technique, while all patients in our study received IMRT.

Intriguingly, some studies have suggested that a higher splenic dose is linked to a reduced risk of grade 3–4 leukopenia. One possible mechanism is that irradiation leads to the release of sequestered leukocytes from the spleen. These observations align with the clinical use of splenic irradiation to alleviate leukopenia resulting from hypersplenism [37, 38]. Another plausible explanation is that splenic irradiation may disrupt the balance between leukocyte apoptosis in the spleen and leukocyte production in the bone marrow. Our data reveal that 17.7% (17/96) of patients experienced grade 3–4 leukopenia, a lower incidence compared with the 30% (18/60) observed in Chin *et al*.’s study. This suggests that a higher splenic dose may ameliorate leukopenia following radiotherapy. Neutropenia and lymphopenia are two different conditions of leukopenia. Supportive therapies like recombinant human granulocyte colony-stimulating factor (rhG-CSF) are widely used, which makes neutropenia effectively prevented and controlled in clinical practice. However, treatments for lymphopenia are limited, making prevention extremely important. Although lymphopenia does not lead to life-threatening diseases, many studies have shown that lymphopenia is strongly associated with a poor prognosis, especially in the era of immunotherapy [19]. Therefore, we call for increased focus on RIL in this study.

Previous studies have shown that vertebral body or pelvis exposed to radiation is associated with leukopenia [33, 34]. It is explainable by previously documented radiation-induced functional impairment of the bone marrow stem cells and cell killing of circulating white blood cells [35]. Thus, the severity of leukopenia during radiotherapy is certainly not to be ignored and should be closely monitored. However, we found no evidence of contributions from spine dose to lymphopenia in our study. And two other similar studies came to the same conclusion [31, 36].

Moreover, our investigation revealed that the difference between pre-treatment ALC and Min ALC during radiotherapy, rather than the difference between pre-treatment ALC and post-treatment ALC, was linked to OS. This finding can likely be attributed to the fact that the recovery of lymphocyte counts after CRT does not completely offset the adverse long-term outcomes caused by severe lymphopenia during CRT [39]. Additionally, different subsets of lymphocytes exhibit varying radiosensitivities. For instance, CD19^+^ B lymphocytes were highly radiosensitive but could recover rapidly. CD3^+^ T cells were moderately radiosensitive, and notably, the loss of CD3^+^ CD8^+^ T cells was more significant than that of CD3^+^ CD4^+^ T cells during radiotherapy, indicating that CD8^+^ T cells were relatively radiosensitive compared with their CD4^+^ counterparts [40]. Mature CD3^+^ CD8^+^ T cells play a crucial role in cell-mediated immunity, and radiation-induced cell death of CD3^+^ CD8^+^ T cells was an independent predictor of poor prognosis [41]. Even if newly generated naïve T lymphocytes recover after treatment, they may not be capable of secreting cytokines and effectively targeting tumor cells.

However, in our study, there were no significant differences in prognosis (OS and DFS) between patients with grade 3–4 and grade 0–2 lymphopenia or between patients with grade 4 and grade 0–3 lymphopenia. It is important to note that the lymphocyte count and patient prognosis can also be influenced by disease characteristics (e.g. pathological type and pTNM stage), the use of various supportive therapies during CRT (e.g. G-CSF and GM-CSF) and differences in chemotherapy regimens. Nonetheless, persistent lymphopenia may be associated with tumor recurrence, metastasis, and could impact treatment responses, especially in the era of immunotherapy. Therefore, it is crucial to consider the long-term effects on lymphocytes.

NLR, representing the ratio of ANC to ALC, has been recognized as a significant adverse prognostic factor in advanced gastric cancer patients undergoing postoperative CRT, consistent with numerous prior studies underscoring its superior prognostic value compared with TNM staging [42–47]. Our study aligns with these previous findings, confirming NLR as a significant adverse prognostic factor in advanced gastric cancer patients receiving postoperative CRT.

Given the heterogeneity of abdominal malignancies and the spleen’s anatomical proximity to the target area for gastric cancer radiotherapy, this is one of the few retrospective studies exclusively focusing on patients with locally advanced gastric cancer who underwent adjuvant radiotherapy. All enrolled patients underwent R0 resection and displayed uniform characteristics. Our study emphasizes the importance of monitoring spleen dose–volume parameters, particularly V5, as critical indicators for assessing ALC. Importantly, this study also confirms that spleen V5 can serve as an independent predictor of ALC, and ALC changes are associated with OS and DFS. However, our study has several limitations. Firstly, it is a retrospective clinical study with the inherent limitations of *post hoc* analysis. Additionally, changes in the absolute spleen volume during and after treatment were not evaluated due to a lack of follow-up data. Finally, the study comprises a limited sample size of 96 patients and lacks external validation for the prediction model. Therefore, our conclusions need validation through large prospective clinical studies.

## CONCLUSIONS

Significant predictors in the prediction model for Min ALC during RT were identified, including spleen V5, the number of treatment fractions and primary tumor location. ΔALC was determined to be an important prognostic predictor for patients with certain abdominal tumors. The risk of developing grade ≥3 lymphopenia was found to be associated with the maximum dose of the spleen. Therefore, the spleen should be considered as a routine OAR for dose limitation when treatment plans are designed.

## Abbreviations

CRT: Chemoradiotherapy, RT: Radiotherapy, OAR: Organ-at-risk, ALC: Absolute lymphocyte count, RIL: Radiation-induced lymphopenia, Min ALC: Minimum absolute lymphocyte count, IMRT: Intensity-modulated radiation therapy, LNs: Lymph nodes, Dmax: Maximum dose, Dmean: Mean dose, LVI: Lymphovascular invasion, PNI: Perineural invasion, pTNM: Pathologic tumor-node-metastasis, ANC: Absolute neutrophil count, ΔALC: Change in absolute lymphocyte count, NLR: Neutrophil-to-lymphocyte ratio, HR: Hazard ratio

## Conflict of Interest

The authors declare that they have no competing interests.

## Funding

This work was supported by Suzhou Medical Center (Szlcyxzx202103), the National Natural Science Foundation of China (82171828), the Key R&D plan of Jiangsu Province (Social Development, BE2021652), the Subject construction support project of the Second Affiliated Hospital of Soochow University (XKTJHRC20210011), Wu Jieping Medical Foundation (320.6750.2021-01-12), the special project of ‘Technological Innovation’ project of CNNC Medical Industry Co. Ltd (ZHYLTD2021001), Suzhou Science and Education Health Project (KJXW2021018), Foundation of Chinese Society of Clinical Oncology (Y-pierrefabre202102-0113), Beijing Bethune Charitable Foundation (STLKY0016), Research Projects of China Baoyuan Investment Co. (270004), Suzhou Gusu Health Talent Program (GSWS2022028), Foundation of Chinese Society of Clinical Oncology (Y-XD202002/zb-0015) and Open Project of State Key Laboratory of Radiation Medicine and Protection of Soochow University (GZN1202302).

## Authors’ contributions

**Y. M.**: Investigation, Data Curation, Writing - Original Draft; **Y. K.:** Investigation, Writing - Review & Editing; **S. Z.:** Data Curation; **Y. P.:** Formal analysis; **M. X.:** Validation; **J. Z.:** Visualization; **H. X.:** Investigation; **Z. H.:** Data Curation; **P. X.:** Resources; **J. Q.:** Resources, Data Curation, Supervision; **L. Z.:** Conceptualization, Methodology, Supervision. All authors reviewed the manuscript and approved the final version for submission.

## Availability of data and materials

The datasets used during this study are available from the corresponding author on reasonable request.

## Ethics approval and consent to participate

The study was approved by the ethics committee of The Second Affiliated Hospital of Soochow University. Written informed consent was obtained from all patients for using their data.

## Supplementary Material

Supplementary_Table_S1_rrae023

Supplementary_Table_S2_rrae023

Supplementary_Figure_rrae023
