# Supplementary material for: The relationship between splenic dose and radiation-induced lymphopenia
Source: J Radiat Res. 2024 May 7;65(3):337–49. doi: 10.1093/jrr/rrae023 (PMC11115471; doi:10.1093/jrr/rrae023)
Supplement: Supplementary_Table_S1_rrae023 [file supplementary_table_s1_rrae023.docx]

**Supplementary Table S1**

Selective lymph node station irradiation of different primary tumor sites.

| Primary tumor location | Selected regional lymph nodes |
| --- | --- |
| Upper 1/3 stomach and distal EGJ | No. 110, No. 20, No. 1-3, No. 7-12, No. 16a |
| Middle 1/3 stomach | No. 1-3, No. 5-13, No. 14*, No. 16a |
| Lower 1/3 stomach | No. 3, No. 5-9, No. 11p, No. 12-13, No. 14*, No. 16a |

Abbreviations: EGJ = esophagogastric junction

*When No. 12-13 lymph node metastasis or pancreatic invasion
