# Supplementary material for: The relationship between splenic dose and radiation-induced lymphopenia
Source: J Radiat Res. 2024 May 7;65(3):337–49. doi: 10.1093/jrr/rrae023 (PMC11115471; doi:10.1093/jrr/rrae023)
Supplement: Supplementary_Table_S2_rrae023 [file supplementary_table_s2_rrae023.docx]

**Supplementary Table S2**

Spinal dosimetric factors of the gastric cancer patients who had adjuvant chemoradiation

| Characteristics | Median (range) | Patients categorized by post-treatment ALC | | |
| --- | --- | --- | --- | --- |
|  |  | ALC < 0.5 × 10^9^/L | ALC ≥ 0.5 × 10^9^/L | *P* |
| Dmean of spine (cGy) | 3532 (2178-4046) | 3545 (3023-4046) | 3481 (2178-4001) | 0.486 |
| Dmax of spine (cGy) | 4841 (4595-5319) | 4837 (4595-5232) | 4896 (4697-5319) | 0.248 |
| Spine V5 (%) | 100 (96.27-100) | 100 (99.83-100) | 100 (96.27-100) | 0.676 |
| Spine V10 (%) | 99.79 (91.93-100) | 99.69 (96.81-100) | 99.92 (91.93-100) | 0.589 |
| Spine V15 (%) | 97.90 (85.90-100) | 97.47 (93.89-99.74) | 98.24 (85.90-100) | 0.813 |
| Spine V20 (%) | 94.01 (78.95-98.56) | 94.01 (81.44-98.30) | 93.93 (78.95-98.56) | 0.581 |
| Spine V25 (%) | 88.29 (52.50-96.98) | 89.65 (52.50-95.40) | 87.46 (55.29-96.98) | 0.462 |
| Spine V30 (%) | 77.34 (34.11-92.96) | 79.01 (44.17-92.32) | 76.37 (34.11-92.96) | 0.462 |
| Spine V35 (%) | 56.27 (23.72-87.63) | 57.32 (31.72-87.63) | 55.60 (23.72-78.79) | 0.563 |
| Spine V40 (%) | 32.03 (14.78-53.98) | 32.03 (21.17-53.98) | 30.94 (14.78-52.35) | 0.281 |
| Spine V45 (%) | 12.68 (3.24-31.27) | 13.41 (4.69-31.27) | 12.49 (3.24-26.71) | 0.495 |

Spleen V$\mathcal{x}$: proportion of spleen volume receiving at least $\mathcal{x}$ Gy.
