# Supplementary material for: The relationship between splenic dose and radiation-induced lymphopenia
Source: J Radiat Res. 2024 May 7;65(3):337–49. doi: 10.1093/jrr/rrae023 (PMC11115471; doi:10.1093/jrr/rrae023)

**Supplementary Figure 1** The treatment target of a patient with locally advanced gastric cancer who underwent postoperative adjuvant radiotherapy. Transverse plane (upper left); Model view (upper right); Frontal plane (lower left); Sagittal plane (lower right).


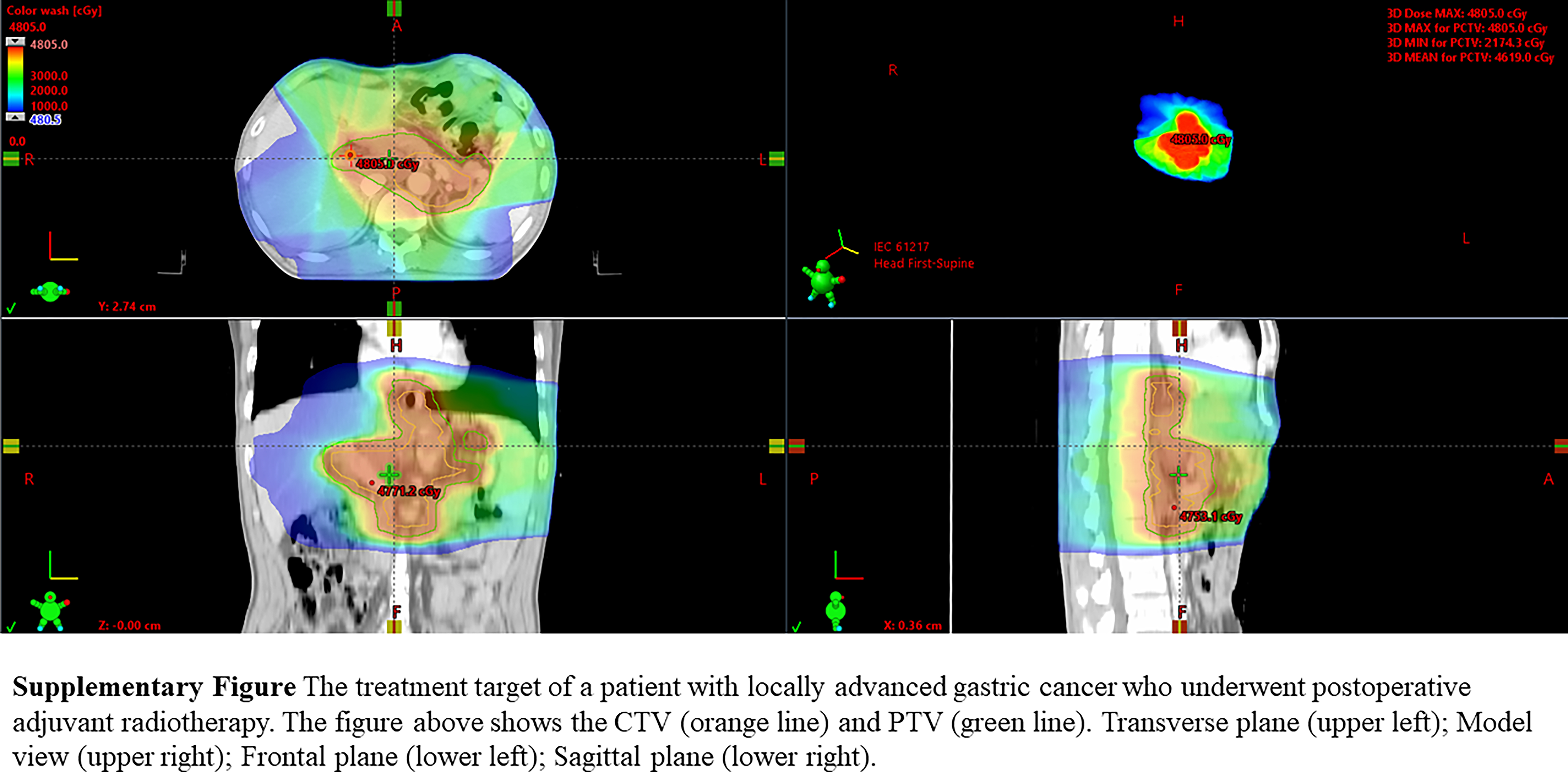

Supplement: Supplementary_Figure_rrae023 [file supplementary_figure_rrae023.docx]
